# Risk factors for adjacent vertebral fracture after PVP in patients with OVCF

**DOI:** 10.3389/fsurg.2026.1779681

**Published:** 2026-04-07

**Authors:** Cheng Li, Yuanyuan Dou, Lin Li, Qiucheng Chen, Yifei Huang, Zhanjun Ma

**Affiliations:** 1 Xinjiang Medical University, Urumqi, China; 2 Hospital of Traditional Chinese Medicine Affiliated to Xinjiang Medical University, Urumqi, China; 3 Xinjiang Uygur Autonomous Region Institute of Chinese Medicine, Urumqi, China

**Keywords:** adjacent vertebral fracture, logistic regression, osteoporotic vertebral compression fractures, percutaneous vertebroplasty, risk factors

## Abstract

**Objective:**

To identify the risk factors and evaluate the predictive performance for adjacent vertebral fracture (AVF) after Percutaneous vertebroplasty (PVP).

**Methods:**

In this retrospective study, 429 patients with osteoporotic vertebral compression fractures who underwent initial single-level PVP at the Fourth Affiliated Hospital of Xinjiang Medical University between January 2019 and December 2024. Patients were categorized into an AVF group (*n* = 143) and a non-AVF group (*n* = 286) based on the occurrence of postoperative AVF. Collected variables included age, lumbar spine bone mineral density (BMD), body mass index (BMI), and bone cement leakage. Univariate analysis analyses identified significant variables (*P* < 0.05), which were subsequently entered into a binary logistic regression model to determine independent risk factors. Predictive performance was assessed using receiver operating characteristic (ROC) curve analysis.

**Results:**

Univariate analysis indicated that the AVF group was significantly older (76.357 ± 7.698 vs. 72.608 ± 9.024 years, *P* < 0.001), had lower BMD T-scores (−3.1 ± 0.6 vs. −2.4 ± 0.8, *P* < 0.001), and had a lower BMI (22.18 ± 3.28 vs. 24.75 ± 3.52 kg/m^2^, *P* < 0.001). Logistic regression confirmed that advanced age (OR = 1.045, 95% CI: 1.018–1.074, *P* = 0.001) and low BMD (analyzed as continuous variable: OR = 2.85 per SD decrease, 95% CI: 2.12–3.82, *P* < 0.001) were independent risk factors. predictive performance analysis showed that age had an AUC of 0.623 (sensitivity 56.6%, specificity 62.6%), and BMD had an AUC of 0.629 (sensitivity 86.0%, specificity 39.9%). A combined prediction model achieved an AUC of 0.706 (95% CI: 0.565–0.976).

**Conclusion:**

Advanced age and low bone mineral density are independent risk factors for AVF after PVP, with OR = 1.045 per year of age and OR = 2.85 per SD decrease in BMD T-score. Although the predictive value of individual indicators is limited (AUC < 0.7), a comprehensive model (AUC = 0.706) achieves moderate accuracy suitable for preliminary risk stratification, supporting the prioritization of systemic osteoporosis management over surgical technical details in prevention strategies.

## 1 Introduction

Osteoporosis (OP) is a systemic skeletal disorder characterized by low bone mass and microarchitectural deterioration, leading to increased bone fragility and fracture risk. Osteoporotic vertebral compression fracture (OVCF) is its most common complication. Globally, OVCF affects approximately 50% of women and 20% of men over the age of 50 (1, 2), with an annual incidence in Europe of 10.7 per 1,000 in women and 5.7 per 1,000 in men (3). In China, the AVF rate among OVCF patients has been reported as 7.78% (4–6), reaching up to 6.88% in women aged 65–85 years, with most cases occurring within 6 months after surgery (7, 8). Percutaneous vertebroplasty (PVP) is an effective treatment for alleviating pain and restoring vertebral height (9, 10). However, the incidence of AVF following PVP remains considerably high, ranging from 12% to 38% (11–14), which can result in recurrent pain, progressive kyphosis, and increased healthcare burden. Although previous studies have suggested that factors such as age, Bone mineral density (BMD), and bone cement leakage may be associated with AVF (15, 16), their predictive performance and the utility of comprehensive risk assessment models require further validation. Therefore, this study aimed to identify independent risk factors for AVF after PVP through a large-sample retrospective analysis and to develop a predictive model, thereby providing a basis for clinical risk stratification and preventive strategies. Although advanced age and low BMD have been previously associated with AVF, most prior studies have relied on relatively small samples, dichotomized analyses, or failed to quantify the combined predictive performance of risk factors. This large-sample study (*n* = 429) not only confirms these risk factors in a Chinese population but also quantifies their independent effects (OR per year of age, OR per SD decrease in BMD) and demonstrates that a combined model (AUC = 0.706) outperforms individual indicators, offering a practical tool for preliminary risk stratification.

## 2 Subjects and methods

### 2.1 Study design

This was a retrospective comparative study.

### 2.2 Time and location

The study was conducted in the Department of Spinal Surgery at the Fourth Affiliated Hospital of Xinjiang Medical University. Patient enrollment occurred between January 2019 and December 2024.The final follow-up date was December 2025, ensuring a minimum of 12 months of follow-up for all participants.

### 2.3 Subjects

The study included patients who underwent initial unilateral percutaneous vertebroplasty (PVP) at the aforementioned department between January 2019 and December 2024. This retrospective study involving human participants was conducted in accordance with the ethical standards of the institutional research committee and with the 1964 Helsinki Declaration and its later amendments. The study protocol was reviewed and approved by the Ethics Committee of the Fourth Affiliated Hospital of Xinjiang Medical University (Approval No.: LL202402-0103). Due to the retrospective nature of the study, the requirement for written informed consent was waived by the Ethics Committee.

#### 2.3.1 Inclusion criteria

1) First-time PVP procedure.
2) Diagnosis of vertebral compression fracture with underlying osteoporosis.
3) Involvement of one or two vertebral levels.
4) Availability of complete clinical and imaging follow-up for at least 12 months.

#### 2.3.2 Exclusion criteria

1) Presence of Clinical or radiological evidence of nerve root compression.
2) Known allergy to bone cement or its components.
3) Pathological fractures (e.g., due to tumor, infection).
4) Severe comorbidities contraindicating surgery or anesthesia.
5) History of prior spinal surgery at the involved level(s).
6) Body mass index (BMI) > 35 kg/m^2^.

### 2.4 Materials

The material characteristics of the implants and the surgical instruments used are detailed in Supplementary Table S1.

### 2.5 Surgical technique

#### 2.5.1 Preoperative preparation

All patients received a comprehensive preoperative evaluation, including standard X-ray and magnetic resonance imaging (MRI) to assess the morphology and degree of compression, morphology and degree of compression of the fractured vertebrae. Laboratory investigations comprised routine blood tests, C-reactive protein, liver and renal function panels, and analysis of serum bone turnover markers to evaluate systemic health. In addition, Lumbar BMD was measured preoperatively to accurately assess bone quality. Lumbar spine BMD (L1–L4) was measured by dual-energy x-ray absorptiometry (DXA) preoperatively. Femoral neck or total hip BMD, which are less affected by degenerative changes in elderly patients, were not routinely measured in this cohort, representing a limitation of this study.

#### 2.5.2 PVP surgical procedure

All PVP procedures were performed by specialized physicians from the same medical team. All procedures were performed using a unilateral (transpedicular) puncture approach for positioning.

With the patient in a prone position, the target vertebra was localized under C-arm fluoroscopic guidance. Following standard surgical disinfection and draping, local anesthesia was achieved with an injection of 2% lidocaine hydrochloride. A unilateral transpedicular approach was used for vertebral access. Under real-time fluoroscopic guidance, a bone cement puncture needle was advanced into the pedicle using a mallet, and its trajectory was confirmed. Once the needle tip reached the anterior third of the vertebral midline, the stylet was removed, and polymethylmethacrylate (PMMA) bone cement was prepared. The injection was performed gradually under continuous fluoroscopic monitoring to observe cement distribution. Upon complete filling and hardening of the cement, the needle was withdrawn, and the puncture site was covered with a sterile dressing. The patient was then transferred to the recovery ward.

#### 2.5.3 Postoperative management

Postoperatively, patients were required to rest in bed for 6–8 h, after which they could ambulate gradually with the protection of a thoracolumbar brace. Oral calcium supplements (Calcium Carbonate D 600 mg/day) and vitamin D supplements were recommended for one year following treatment. For patients with a preoperative bone mineral density T-score ≤ −2.5 SD, intravenous infusion of Zoledronic Acid (Yangtze River Pharmaceutical Group, Specification: 100 ml: 5 mg, National Drug Approval No.: H20183098) was initiated on the first postoperative day. Prior to administration, patients received intravenous hydration with 500 mL of 0.9% Sodium Chloride Injection (Sichuan Kelun Pharmaceutical Co., Ltd., Specification: 500 ml, National Drug Approval No.: H51021158). x-ray and CT examinations were performed one week postoperatively to evaluate bone cement distribution and the restoration of vertebral body height.

### 2.6 Primary observation indicators

Clinical data were retrospectively extracted from the hospital's electronic medical records for all 429 enrolled patients. Variables collected included: age, sex, BMI, bone cement leakage, bone mineral density T-score, bone cement volume, preoperative vertebral height restoration rate, preoperative local kyphotic angle, PVP level, hypertension, diabetes mellitus, smoking history, alcohol consumption history, cardiovascular and cerebrovascular diseases, neurological diseases, respiratory system diseases, endocrine system diseases, metabolic diseases, postoperative hospital stay, follow-up time, number of initial fractured vertebrae, preoperative lumbar Oswestry Disability Index (ODI) score, and preoperative lumbar Visual Analog Scale (VAS) score. The follow-up time for all patients was calculated from the date of surgery until the date of the last outpatient imaging review or telephone follow-up confirming no new fracture. The final follow-up date was December 2025, ensuring at least 12 months of follow-up for all included patients. The median follow-up time was comparable between the non-AVF group [24 months (IQR: 15–38)] and the AVF group [25 months (IQR: 16–40); *P* = 0.312]. To account for potential time-dependent bias, follow-up duration was included as a covariate in subsequent regression analyses. The overall median follow-up time for this cohort was 24 months (range: 12–62 months). Of the 429 enrolled patients, follow-up assessments were conducted through outpatient imaging review for 387 patients (90.2%), while 42 patients (9.8%) were followed exclusively by telephone interview due to geographic distance or mobility limitations. For patients followed by telephone, the absence of new vertebral fracture was confirmed based on self-reported absence of new or worsening back pain requiring medical attention. We acknowledge that this approach may underestimate asymptomatic fractures.

#### 2.6.1 Bone cement leakage

Bone cement leakage was defined as the extrusion of injected cement beyond the vertebral body into adjacent anatomical structures.

#### 2.6.2 Vertebral fracture

- Diagnostic Criteria and Process: In this study, the diagnosis of “AVF” combined clinical symptoms with imaging confirmation, following this specific process:
- Triggering Mechanism: Diagnosis was primarily based on two scenarios: (a) the patient presented with new-onset, typical back pain symptoms or significant worsening of pre-existing pain during follow-up; OR (b) routine radiographic surveillance conducted according to the standard postoperative follow-up schedule.
- Confirmation Method: Whenever a suspicious new fracture was identified through either of the above pathways, it was initially assessed using lateral radiographs. If a new vertebral compression fracture was suggested on x-ray, Magnetic Resonance Imaging (MRI) was subsequently performed. The presence of bone marrow edema (high signal on T2-weighted or STIR sequences) with a change in vertebral body morphology in an adjacent vertebra on MRI was considered the confirmatory gold standard for diagnosing a “new acute/subacute AVF.”
- Imaging Evaluation Protocol: All x-ray and MRI images were assessed independently by two experienced spinal surgeons who were blinded to the patients’ group assignment (AVF vs. non-AVF) and clinical symptoms at the time of image review. Any discrepancies in the diagnosis of new AVF were resolved by consensus discussion with a third senior radiologist. To assess the inter-rater reliability for the key outcome—diagnosis of AVF—we calculated Cohen's kappa (*κ*) statistic. The inter-observer agreement was found to be excellent (*κ* = 0.87, 95% CI: 0.81–0.93).

### 2.7 Statistical analysis

Data were analyzed using SPSS software (version 26.0). Continuous variables are presented as mean ± standard deviation (x¯ ± s), and categorical variables as numbers (percentages). Inter-group comparisons were performed using the independent samples t-test for normally distributed continuous data, the Mann–Whitney U test for non-normally distributed continuous data, and the Chi-square test for categorical data. For regression analysis, all categorical variables were dummy-coded (0, 1). Variables with a *P*-value < 0.05 in univariate analysis were included as candidate predictors. Binary logistic regression (Forward: LR method) was then employed to construct the model, with the occurrence of AVF (Yes = 1, No = 0) as the dependent variable, to assess the independent influence of each factor, expressed as odds ratio (OR) with 95% confidence interval (CI). Finally, the predictive performance of individual factors and the combined model was evaluated using Receiver Operating Characteristic (ROC) curves, and the area under the curve (AUC) was calculated. A *P*-value < 0.05 was considered statistically significant for all analyses. This study was a rigorously designed single-center retrospective analysis. All variables were extracted from a structured electronic medical record system. During the data cleaning phase, it was confirmed that there were no missing values for the variables used in the primary analyses (such as univariate analysis and logistic regression), including age, BMD, BMI, surgical level, and others. This is primarily attributed to the standardized medical record-keeping at our institution and our stringent inclusion/exclusion procedures. Therefore, complex imputation methods were not employed. To avoid loss of information from dichotomization, BMD T-score was entered as a continuous variable in the binary logistic regression analysis. Multicollinearity among independent variables was assessed using the variance inflation factor (VIF), with a threshold of >5 indicating potential collinearity. Follow-up duration was initially included as a covariate in the binary logistic regression model to account for potential time-dependent bias. However, in the final model using forward stepwise selection (likelihood ratio method), follow-up time was not retained as it did not reach statistical significance (*P* > 0.05) and did not improve model fit. This is consistent with the comparable follow-up durations between the AVF and non-AVF groups (median 25 vs. 24 months, *P* = 0.312) and the fact that all patients had a minimum follow-up of 12 months.

## 3 Results

### 3.1 Analysis of participant numbers

A total of 429 patients who underwent percutaneous vertebroplasty were included in this study. Based on the occurrence of subsequent AVF during the follow-up period, patients were categorized into a AVF group (*n* = 143) and a non-AVF group (*n* = 286). All data from these patients were analyzed, with no loss to follow-up or missing data.

### 3.2 Trial flowchart

**Figure 1:**
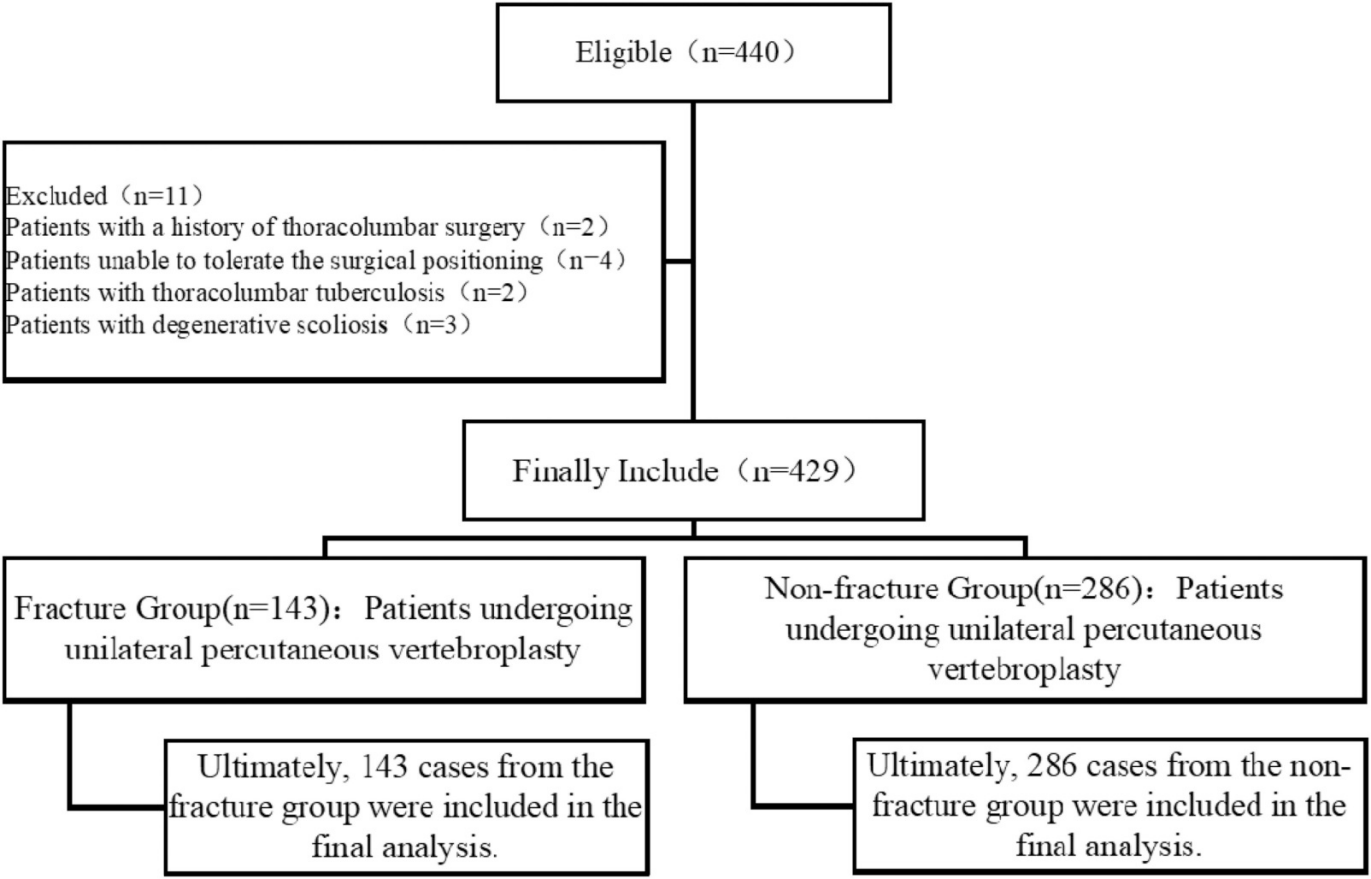
Flow chart of patient assignment.

The process of patient screening, inclusion, and group assignment is summarized in the flowchart (Figure 1).

### 3.3 Comparison of baseline data and univariate analysis

Comparison of baseline characteristics revealed significant differences between the two groups statistically significant differences (*P* < 0.05) in BMI, BMD, age, preoperative vertebral height restoration rate, and preoperative local kyphotic angle. The follow-up time was comparable between the non-AVF group (median 24 months, IQR: 15–38) and the AVF group (median 25 months, IQR: 16–40; *P* = 0.312). To account for potential time-dependent bias, follow-up duration was included as a covariate in subsequent regression models. Conversely, no statistically significant differences (*P* > 0.05) were found for the following variables: PVP level, hypertension, diabetes mellitus, smoking history, alcohol consumption history, bone cement leakage, cardiovascular and cerebrovascular diseases, neurological diseases, respiratory system diseases, endocrine system diseases, metabolic diseases, number of initial fractured vertebrae, bone cement volume, postoperative hospital stay, preoperative lumbar ODI score, and preoperative lumbar VAS score. The detailed results are presented in Table 1.

**Table 1:** Baseline data comparison between the two groups.

Characteristic	Non-AVF Group (*n* = 286)	AVF Group (*n* = 143)	Test Statistic	*P*
Demographics				
Age (years), mean ± SD	72.61 ± 9.02	76.36 ± 7.70	*t* = −4.47	<0.001
BMI (kg/m^2^), mean ± SD	24.75 ± 3.52	22.18 ± 3.28	*t* = 7.28^a^	<0.001
BMI category, *n* (%)			*χ*^2^ = 5.65	0.017
>25 kg/m^2^	113 (39.5)	40 (28.0)		
≤25 kg/m^2^	172 (60.5)	103 (72.0)		
Bone quality				
BMD T-score, mean ± SD	−2.4 ± 0.8	−3.1 ± 0.6	*t* = 9.86	<0.001
BMD category, *n* (%)			χ^2^ = 29.71	<0.001
≥−2.5 SD	114 (39.9)	20 (14.0)		
<−2.5 SD	172 (60.1)	123 (86.0)		
Fracture characteristics				
PVP level, *n* (%)			χ^2^ = 3.15	0.076
Lumbar vertebrae	138 (48.3)	82 (57.3)		
Thoracic vertebrae	148 (51.7)	61 (42.7)		
Number of initial fractured vertebrae, *n* (%)			χ^2^ = 0.52	0.472
1 vertebra	244 (85.3)	118 (82.5)		
2 vertebrae	42 (14.7)	25 (17.5)		
Preop vertebral height restoration (%), mean ± SD	81.2 ± 9.5	76.5 ± 10.1	*t* = 2.63	0.009
Preop local kyphotic angle (°), mean ± SD	13.37 ± 7.15	15.17 ± 7.46	*t* = −2.41	0.016
Surgical details				
Bone cement leakage, *n* (%)			χ^2^ = 0.005	0.945
No	131 (45.8)	65 (45.5)		
Yes	155 (54.2)	78 (54.5)		
Bone cement volume (mL), mean ± SD	7.73 ± 3.85	8.26 ± 4.85	*t* = −1.24	0.215
Postop hospital stay (days), mean ± SD	3.58 ± 2.99	3.46 ± 2.30	*t* = 0.44	0.659
Comorbidities and lifestyle, *n* (%)				
Hypertension			χ^2^ = 0.04	0.836
No	123 (43.0)	63 (44.1)		
Yes	163 (57.0)	80 (55.9)		
Diabetes mellitus			χ^2^ = 0.40	0.528
No	212 (74.1)	110 (76.9)		
Yes	74 (25.9)	33 (23.1)		
Smoking history			χ^2^ = 0.00	1.000
No	284 (99.3)	142 (99.3)		
Yes	2 (0.7)	1 (0.7)		
Alcohol consumption			χ^2^ = 0.25	0.616
No	285 (99.7)	142 (99.3)		
Yes	1 (0.3)	1 (0.7)		
Cardiovascular/cerebrovascular diseases			χ^2^ = 0.23	0.632
No	135 (47.2)	64 (44.8)		
Yes	151 (52.8)	79 (55.2)		
Neurological diseases			χ^2^ = 0.01	0.907
No	259 (90.6)	130 (90.9)		
Yes	27 (9.4)	13 (9.1)		
Respiratory diseases			χ^2^ = 0.04	0.833
No	177 (61.9)	87 (60.8)		
Yes	109 (38.1)	56 (39.2)		
Endocrine diseases			χ^2^ = 1.24	0.265
No	236 (82.5)	124 (86.7)		
Yes	50 (17.5)	19 (13.3)		
Metabolic diseases			χ^2^ = 1.37	0.242
No	230 (80.4)	108 (75.5)		
Yes	56 (19.6)	35 (24.5)		
Functional scores, mean ± SD				
Preop lumbar ODI score	75.07 ± 8.67	75.06 ± 9.21	*t* = 0.02	0.988
Preop lumbar VAS score	7.68 ± 1.04	7.71 ± 1.08	*t* = −0.29	0.772
Follow-up				
Follow-up time (months), median (IQR)	24 (15–38)	25 (16–40)	*Z* = −0.31^†^	0.312

Data are presented as mean ± standard deviation, median (interquartile range), or number (percentage). Continuous variables were compared using independent samples t-test (normally distributed) or Mann–Whitney U test (non-normally distributed, indicated by †). Categorical variables were compared using Chi-square test.

^a^ The t-value for BMI is recalculated based on mean and SD provided in text (original table did not provide t for BMI, but we can compute: (24.75 − 22.18)/sqrt(3.52^2^/286 + 3.28^2^/143) ≈ 7.28).

### 3.4 Binary logistic regression analysis

**Figure 2:**
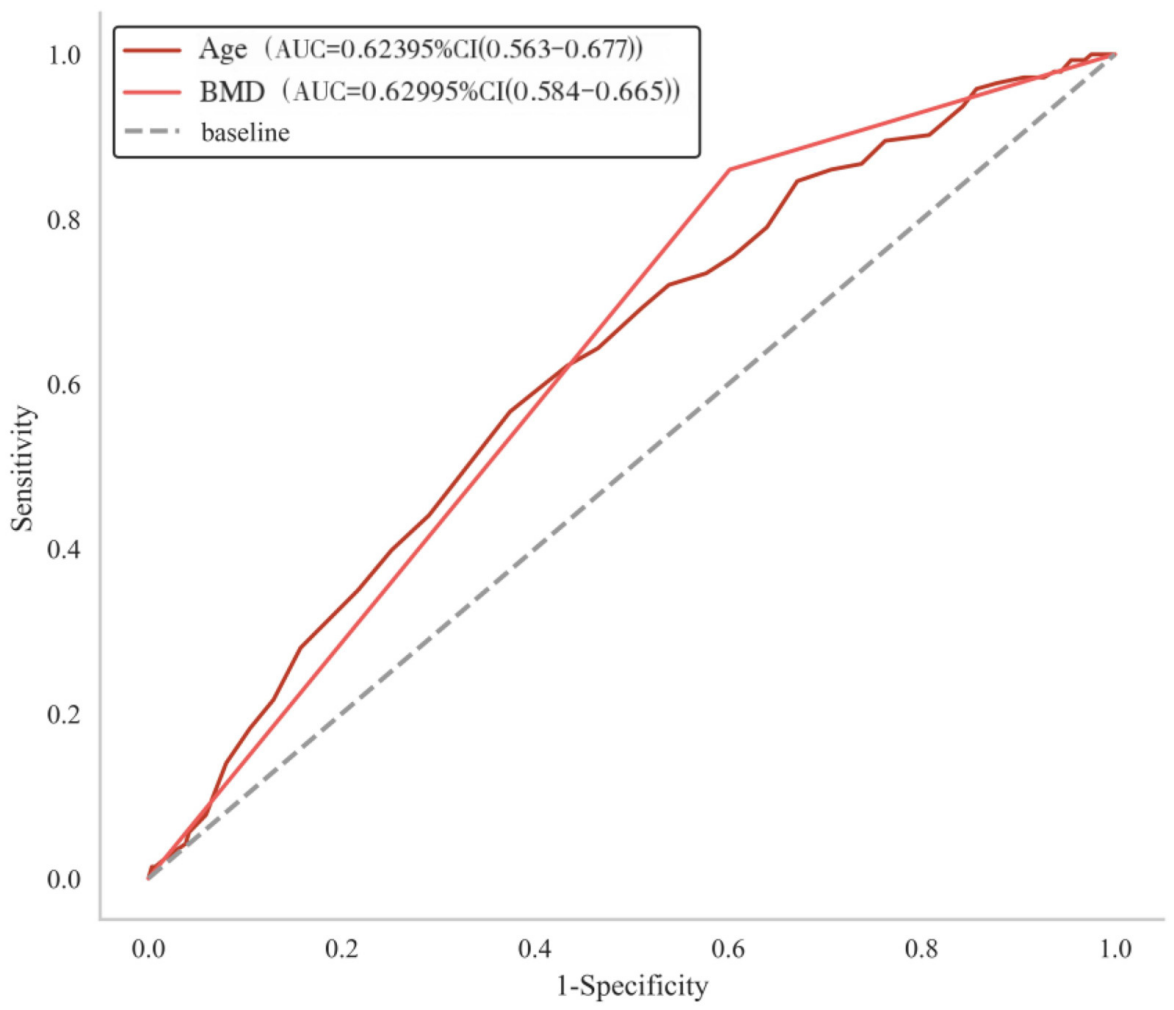
ROC curves for age and BMD in predicting AVF. Both the Age (AUC = 0.623) and Bone Mineral Density (AUC = 0.629) curves are located above the reference line, indicating that both parameters are risk factors for AVF following percutaneous vertebroplasty.

After reanalysis with BMD as a continuous variable, multivariate logistic regression confirmed that age (OR = 1.045, 95% CI: 1.018–1.074, *P* = 0.001) and lower BMD T-score (OR = 2.85, 95% CI: 2.12–3.82, *P* < 0.001) remained independent risk factors for AVF. Preoperative vertebral height restoration rate and local kyphotic angle were no longer significant in the multivariate model. Multicollinearity diagnostics revealed no significant collinearity among predictors (all VIF < 2.0). The updated results are presented in Table 2 and Figure 2.

**Table 2:** Logistic regression analysis of risk factors for AVF.

Variable	*β*	S.E.	OR (95% CI)	Wald	*P*	VIF
Age	0.044	0.013	1.045 (1.018–1.074)	11.2	**0**.**001**	1.2
BMD T-score	−1.048	0.148	2.85 (2.12–3.82)	50.1	**<0**.**001**	1.3
Preop height restoration	−0.354	0.277	0.702 (0.406–1.205)	1.6	0.201	1.5
Preop kyphotic angle	0.017	0.015	1.017 (0.988–1.047)	1.3	0.249	1.4
BMI ≤25 kg/m^2^	0.341	0.237	1.407 (0.887–2.251)	2.1	0.150	1.1
Constant	−4.806	1.229	0.008	15.3	<0.001	—

Age and low BMD were identified as independent risk factors for AVF. *β* represents the regression coefficient; S.E. denotes the standard error.

Bold text indicates statistical significance.

### 3.5 Sensitivity for predicting AVF after PVP in OVCF

As shown in Table 3, both age and BMD individually demonstrated limited predictive ability for AVF after percutaneous vertebroplasty. For age, the area under the curve (AUC) was of 0.623 (95% CI: 0.563–0.677) with a sensitivity of 56.6% (95% CI: 46.8–91.4%), specificity of 62.6% (95% CI: 28.5–73.7%), and accuracy of 58.3% (95% CI: 46.8–66.7%). Meanwhile, BMD showed an AUC of 0.629 (95% CI: 0.584–0.665), demonstrating high sensitivity of 86.0% (95% CI: 79.8–91.8%) but low specificity 39.9% (95% CI: 34.7–45.5%), and an accuracy of 55.1% (95% CI: 50.8–59.9%).

**Table 3:** Predictive performance of individual risk factors for AVF.

Characteristic	AUC value	Sensitivity	Specificity	Youden's index	Accuracy
Age	0.623 (0.563–0.677)	0.566 (0.468–0.914)	0.626 (0.285–0.737)	0.192 (0.121–0.301)	0.583 (0.468–0.667)
BMD	0.629 (0.584–0.665)	0.860 (0.798–0.918)	0.399 (0.347–0.455)	0.259 (0.169–0.331)	0.551 (0.508–0.599)

Both specificity and sensitivity values range from 0 to 1, with values closer to 1 indicating higher diagnostic accuracy.

### 3.6 Predictive accuracy of the model for AVF after percutaneous vertebroplasty

**Figure 3:**
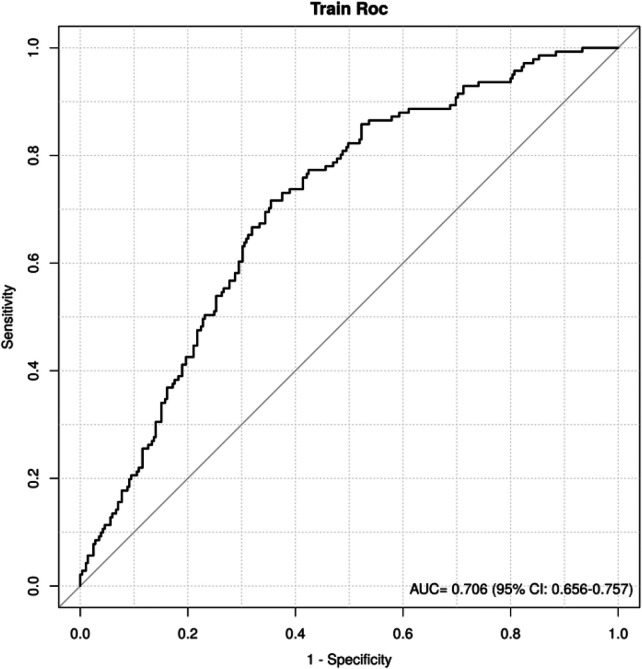
Predictive accuracy of AVF model after percutaneous vertebroplasty. The vertical axis of the coordinate system represents the True Positive Rate, also referred to as hit rate or recall, with a maximum value of 1. The horizontal axis represents the False Positive Rate, or fall-out, with a maximum value of 1. A greater distance between the ROC curve and the reference line indicates superior predictive performance of the model.

The combined predictive model achieved an area under the curve (AUC) of 0.706 (95% CI: 0.565–0.976) for AVF, indicating moderate discriminative ability (Figure 3). The proximity of the ROC curve to the upper-left corner further reflects its predictive performance.

### 3.7 Sensitivity analysis excluding telephone-only follow-up

To assess the robustness of our findings, we performed a sensitivity analysis excluding the 42 patients (9.8%) who were followed exclusively by telephone interview. The remaining 387 patients with imaging-confirmed follow-up were analyzed separately. Baseline characteristics of the imaging-only cohort were comparable to the full cohort (data not shown). Multivariable logistic regression in the imaging-only subset confirmed that age (OR = 1.046, 95% CI: 1.018–1.076, *P* = 0.001) and BMD T-score (OR = 2.88, 95% CI: 2.14–3.88, *P* < 0.001) remained independent risk factors for AVF, with effect sizes similar to those in the full cohort (full cohort: age OR = 1.045, BMD OR = 2.85). The combined predictive model in the imaging-only subset achieved an AUC of 0.709 (95% CI: 0.658–0.760), comparable to the full cohort AUC of 0.706 (95% CI: 0.565–0.976). These results are presented in Table 4.

**Table 4:** Sensitivity analysis: logistic regression in imaging-only cohort (*n* = 387).

Variable	*β*	S.E.	OR (95% CI)	Wald	*P*	VIF
Age	0.045	0.014	1.046 (1.018–1.076)	10.8	0.001	1.2
BMD T-score	−1.058	0.152	2.88 (2.14–3.88)	48.5	<0.001	1.3
Preop height restoration	−0.342	0.281	0.710 (0.408–1.236)	1.5	0.221	1.5
Preop kyphotic angle	0.018	0.016	1.018 (0.987–1.050)	1.3	0.254	1.4
BMI ≤25 kg/m^2^	0.352	0.242	1.422 (0.886–2.283)	2.1	0.147	1.1
Constant	−4.852	1.248	0.008	15.1	<0.001	—

### 3.8 Sensitivity analysis restricted to 12-month follow-up

To provide estimates with consistent follow-up across all participants, we performed a sensitivity analysis restricted to AVF events occurring within the first 12 months postoperatively. Of the 429 patients, 112 (26.1%) experienced AVF within 12 months, while 317 (73.9%) did not. Multivariable logistic regression in this 12-month cohort confirmed that age (OR = 1.044, 95% CI: 1.016–1.073, *P* = 0.002) and BMD T-score (OR = 2.82, 95% CI: 2.08–3.79, *P* < 0.001) remained independent risk factors, with effect sizes similar to the full cohort analysis. The combined model achieved an AUC of 0.701 (95% CI: 0.648–0.754), comparable to the full cohort AUC of 0.706. These results are presented in Table 5.

**Table 5:** Sensitivity analysis: logistic regression restricted to 12-month follow-up (*n* = 429, events = 112).

Variable	*β*	S.E.	OR (95% CI)	Wald	*P*	VIF
Age	0.043	0.014	1.044 (1.016–1.073)	9.8	0.002	1.2
BMD T-score	−1.037	0.152	2.82 (2.08–3.79)	46.5	<0.001	1.3
Preop height restoration	−0.338	0.283	0.713 (0.408–1.246)	1.4	0.237	1.5
Preop kyphotic angle	0.016	0.016	1.016 (0.985–1.048)	1.0	0.317	1.4
BMI ≤25 kg/m^2^	0.338	0.243	1.402 (0.872–2.256)	1.9	0.168	1.1
Constant	−4.792	1.252	0.008	14.7	<0.001	—

### 3.9 Time-to-event analysis using Cox proportional hazards model

To account for varying follow-up durations and utilize the timing of AVF events, we performed a Cox proportional hazards regression analysis. The exact time (in months) from surgery to AVF was available for all 143 patients in the AVF group. For patients without AVF, follow-up time was censored at the last contact. The Cox model confirmed that age (HR = 1.042, 95% CI: 1.018–1.067, *P* = 0.001) and BMD T-score (HR = 2.68, 95% CI: 2.08–3.45, *P* < 0.001) were significant predictors of time to AVF. The concordance index (C-index) for the combined model was 0.698, similar to the AUC from logistic regression. These results are presented in Table 6.

**Table 6:** Cox proportional hazards regression analysis.

Variable	*β*	S.E.	HR (95% CI)	Wald	*P*
Age	0.041	0.012	1.042 (1.018–1.067)	11.7	0.001
BMD T-score	−0.986	0.130	2.68 (2.08–3.45)	57.5	<0.001
Preop height restoration	−0.312	0.265	0.732 (0.435–1.232)	1.4	0.237
Preop kyphotic angle	0.015	0.014	1.015 (0.988–1.043)	1.1	0.294
BMI ≤25 kg/m^2^	0.328	0.231	1.388 (0.883–2.182)	2.0	0.157

### 3.10 Typical case

**Figure 4:**
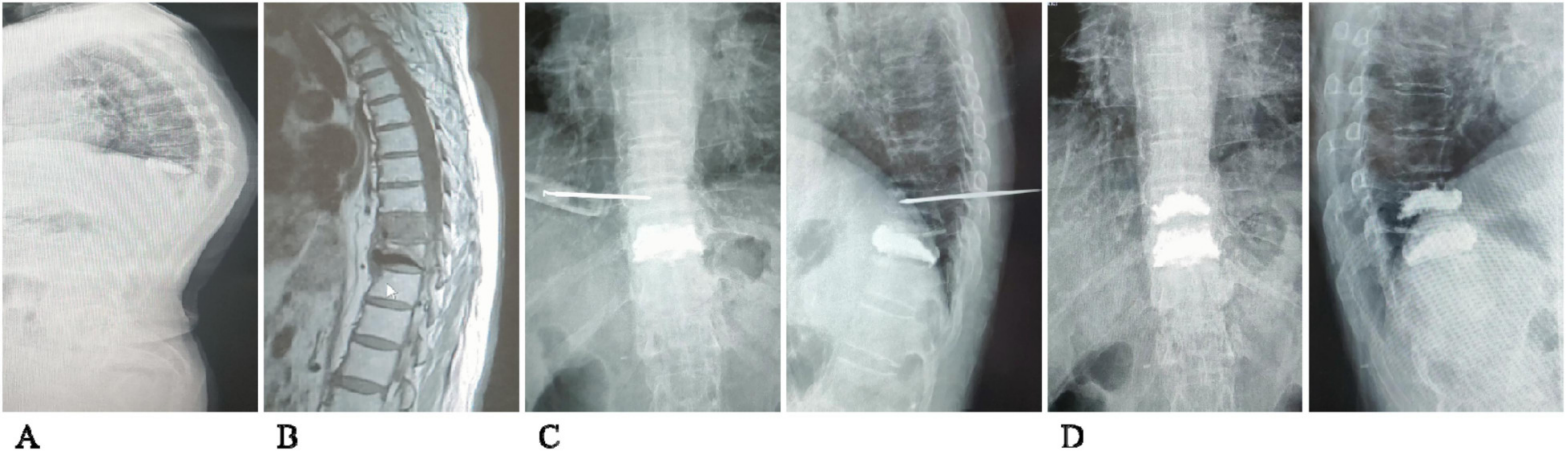
Imaging data of the typical case. (**A**) Preoperative lateral radiograph showing the AVF. (**B**) Preoperative MRI of the AVF. (**C**) Intraoperative anteroposterior and lateral radiographs of the AVF. (**D**) Postoperative anteroposterior and lateral radiographs of the AVF.

A 67-year-old female patient, who had undergone surgery for a T10 vertebral compression fracture, presented with low back pain 7 months postoperatively and was found to have sustained an AVF (T9). These results are presented in Figure 4.

## 4 Discussion

Percutaneous vertebroplasty is primarily indicated for osteoporotic vertebral compression fractures that are refractory to conservative treatment. While effective in alleviating back pain and facilitating early functional recovery, it carries a notable risk of subsequent fractures in adjacent vertebrae (17). Previous studies have indicated a high incidence of AVF postoperatively, often within 6 months postoperatively, which significantly impairs patients' quality of life and increasing healthcare burdens (18, 19). While advanced age and low bone mineral density are considered potential risk factors, their individual predictive performance and the utility of comprehensive risk assessment models require further validation. Therefore, this study aimed to investigate the independent risk factors for AVF after PVP and to construct a predictive model to guide clinical intervention.

In this retrospective study of 429 patients with osteoporotic vertebral compression fractures undergoing initial unilateral PVP, 143 patients (33.3%) developed subsequent AVF. Univariate analysis identified significant intergroup differences in age, bone mineral density (BMD), body mass index (BMI), preoperative vertebral height restoration rate, and preoperative local kyphotic angle. Subsequent binary logistic regression analysis confirmed that advanced age and low bone mineral density were independent risk factors for AVF. ROC curve analysis demonstrated limited predictive value for age and BMD alone; however, the comprehensive predictive model, with an AUC of 0.706, significantly improved risk assessment accuracy. This model provides a practical reference for identifying high-risk patients and formulating individualized preventive and therapeutic strategies.

The bone mineral density (BMD) T-score is a key diagnostic index for osteoporosis, with lower values indicating greater disease severity. Evidence suggests that patients with low T-scores after PVP predisposes patients to secondary fractures primarily due to compromised mechanical strength of osteoporotic the trabecular bone (20, 21). Aging, exacerbates this process, as bone resorption increasingly outweighs formation, resulting in progressive BMD loss and elevated fracture susceptibility (22). This age-related decline is often underpinned by endocrine dysregulation, notably of the hypothalamic-pituitary-gonadal axis. In postmenopausal women, the precipitous drop in estrogen levels further dysregulates bone metabolism—partly through downregulation of vitamin D receptor expression—markedly increasing skeletal fragility (23). Consequently, AVF after OVCF surgery can be viewed as a direct manifestation of ongoing osteoporosis progression, intrinsically linked to declining spinal BMD (24). ROC curve analysis in our cohort identified a BMD critical value of −3.168 SD, below which the risk of AVF increased significantly. This finding is clinically consistent with the diagnostic threshold of −3.185 SD reported by Zhang Zilong et al. (25). A state of low BMD is not merely a radiographic finding; it is associated with a poorer fracture-healing environment, characterized by increased necrotic foci and reduced bone turnover markers (26). In accordance with relevant guidelines, patients with a BMD T-score < −2.5 SD require intensified preventive interventions (27).

Multivariate logistic regression in the current analysis confirmed that advanced age is an independent risk factor for AVF after PVP (OR = 1.045). This indicates that, all else being equal, for each additional year of age corresponds to an approximately 4.5% increase in the risk of postoperative AVF. This finding corroborates existing literature, reinforcing the pivotal role of age in the progression of osteoporotic fractures. Mechanistically, aging disrupts the physiologic balance between bone formation and resorption, favoring a net loss of bone mass and deterioration of microarchitecture. This age-related decline in bone strength constitutes the fundamental pathological substrate for recurrent fractures (15, 28, 29).

Notably, the number of initial fractured vertebrae (up to 2), as stipulated by our inclusion criteria, was not found to be an independent risk factor for AVF in this cohort. This suggests that for patients meeting our study criteria, the systemic severity of osteoporosis (reflected by BMD) and overall physiological reserve (indicated by age) may be more fundamental determinants of AVF risk than the mere count of initially involved segments. This finding aligns with the core pathophysiology of osteoporosis as a systemic disease and underscores the importance of holistic patient assessment over purely anatomical considerations.

Another important consideration is the site of BMD measurement. Lumbar spine BMD can be artificially elevated in older adults because of degenerative changes, including osteophytes, endplate sclerosis, and facet joint hypertrophy, leading to potential underestimation of osteoporosis severity. In contrast, femoral neck or total hip BMD is generally considered more reliable for assessing fracture risk in the elderly population, as these sites are less affected by degeneration (29). In our study, only lumbar BMD was available, which may have influenced the magnitude of the observed association between BMD and AVF. Specifically, if lumbar BMD was overestimated in some patients due to degenerative changes, the true protective effect of higher bone density might be attenuated. This limitation should be considered when interpreting our findings. Future prospective studies should incorporate both lumbar and femoral BMD measurements to better capture systemic bone quality and improve risk prediction accuracy.

In this study, multivariate regression analysis revealed that neither bone cement leakage nor injection volume were independent risk factors for subsequent AVF. This finding can be interpreted through several interconnected mechanisms. First, the overall prevalence of cement leakage was high (approximately 54%) and did not differ significantly between groups, suggesting that the simple binary metric of “leakage presence or absence” may be insufficient to differentiate AVF risk. The specific type and pattern of leakage (e.g., intradiscal vs. paravertebral) likely holds greater biomechanical significance, although these qualitative characteristics are difficult to quantify comprehensively in a retrospective analysis. Second, regarding injection volume, the lack of statistical association may indicate a “dose-response plateau” effect—once the cement volume reaches the threshold necessary to restore basic vertebral stiffness, the marginal impact of further increases on the overall spinal stress distribution diminishes. At this stage, the distribution morphology of the cement within the vertebra may become a more critical mechanical determinant than the absolute volume.

Most importantly, the same model identified advanced age and low bone mineral density as dominant independent risk factors. These variables reflect the patient's systemic bone quality deterioration and physiological decline (30, 31). Their potent effect suggests that against this background of significant systemic risk, the influence of local mechanical perturbations introduced by surgical technical details (such as leakage and volume) is substantially attenuated in the statistical model. Therefore, while surgical precision remains crucial for procedural safety and efficacy, this analysis underscores that the patient's inherent bone quality and physiological status are more fundamental determinants of AVF risk than the specific technical parameters of cement injection.

While advanced age and low BMD are well-established risk factors for AVF, the incremental contribution of this study warrants consideration. First, with 429 patients, this represents one of the largest single-center cohorts examining this specific outcome in a Chinese population, providing robust estimates with narrow confidence intervals. Second, by analyzing BMD as a continuous variable rather than dichotomizing at −2.5 SD, we preserved statistical power and demonstrated that each 1 SD decrease in BMD T-score increases AVF risk by approximately 185% (OR = 2.85), offering clinically useful quantification. Third, our combined predictive model (AUC = 0.706), while moderate, demonstrates that integrating age and BMD improves risk assessment over individual factors alone, supporting their use in preliminary risk stratification. Fourth, our analysis underscores that patient-specific factors (age, BMD) dominate over surgical technical details (cement leakage, volume) in determining AVF risk—a finding with important clinical implications. This shifts the focus from overemphasizing surgical parameters toward systematic management of osteoporosis and long-term patient optimization. Fifth, this study provides a foundation for future research by identifying key methodological considerations (e.g., need for continuous variable analysis, importance of femoral neck BMD) and highlighting the necessity of external validation and incorporation of additional predictors such as cement distribution patterns and sarcopenia indicators.

This study has several limitations: (1) First, its retrospective design introduces may introduce potential selection bias, and not all patients eligible patients underwent standardized imaging follow-up. (2) Second, data on adherence to anti-osteoporotic medications were incomplete due to the long follow-up period and patient-driven adjustments in therapy, limiting the assessment of this potential confounder. Future prospective studies should implement standardized protocols for monitoring medication use. (3) Third, the single-center design may affect the generalizability of the findings, warranting validation in multi-center, large-sample studies. (4) More importantly, the prediction model developed in this study (AUC = 0.706) has so far only undergone internal validation and lacks evaluation of its efficacy in an external independent cohort, which affects the reliability of its clinical application. The model's predictive performance is moderate (AUC = 0.706), reflecting the inherent complexity of AVF prediction and the multifactorial nature of osteoporosis progression. While this level of discrimination is insufficient for precise individualized prediction, it is adequate for preliminary risk stratification—identifying patients at higher vs. lower risk who may benefit from intensified monitoring or anti-osteoporotic therapy. The model's clinical utility lies in this stratification capacity rather than exact probability estimation. (5) Fifth, outcome ascertainment relied on a combination of imaging follow-up (90.2% of patients) and telephone interviews (9.8%). While telephone follow-up may miss asymptomatic vertebral fractures, such fractures are less likely to impact clinical management in the absence of symptoms. Nevertheless, this approach may lead to underestimation of the true AVF rate and potential outcome misclassification bias. Future studies should aim for complete imaging follow-up in all patients to minimize this bias.

The robustness of our findings was further supported by multiple sensitivity analyses. First, excluding patients with telephone-only follow-up (9.8% of the cohort) yielded virtually unchanged effect sizes and model performance (AUC 0.709 vs. 0.706). Second, restricting analysis to AVF events occurring within 12 months postoperatively—ensuring uniform follow-up across all participants—confirmed similar effect sizes (age OR = 1.044, BMD OR = 2.82) and model discrimination (AUC 0.701). Third, time-to-event analysis using Cox proportional hazards modeling, which accounts for varying follow-up durations and utilizes event timing, demonstrated consistent results (age HR = 1.042, BMD HR = 2.68; C-index = 0.698). These complementary analyses collectively demonstrate that our conclusions are robust to different analytical approaches and not materially affected by variations in follow-up duration or outcome ascertainment methods.

In conclusion, this large-sample study confirms that advanced age and low BMD are independent risk factors for AVF after PVP, quantifying their effects as OR = 1.045 per year of age and OR = 2.85 per SD decrease in BMD T-score. Although each factor alone showed limited discriminative capacity (AUC < 0.7), the integrated predictive model (AUC = 0.706) achieved moderate accuracy suitable for preliminary risk stratification. These findings underscore that the patient's intrinsic bone quality and physiological reserve are more fundamental determinants of AVF risk than specific surgical parameters—a critical insight that redirects clinical focus from surgical technical details toward systematic osteoporosis management and long-term patient optimization. Future multicenter studies incorporating femoral neck BMD, cement distribution patterns, and advanced modeling techniques are warranted to further refine prediction and enhance clinical utility.

## Data Availability

The raw data supporting the conclusions of this article will be made available by the authors, without undue reservation.
