# Supplementary material for: Risk factors for adjacent vertebral fracture after PVP in patients with OVCF
Source: Front Surg. 2026 Apr 7;13:1779681. doi: 10.3389/fsurg.2026.1779681 (PMC13095548; doi:10.3389/fsurg.2026.1779681)
Supplement: Supplementary file 1 [file Table1.docx]

***Material characteristics of the implant***

| Product Designation | Bone Cement（REF66055104） |
| --- | --- |
| **Manufacturer** | Heraeus Medical GmbH |
| **Model** | OSTEOPAL^Ⓡ^V |
| **Authorization Number** | LOT 61185327 |
| **Description and Composition** | Osteopal® V is a rapidly setting, radiopaque bone cement designed for vertebral filling and stabilization, supplied as a two-component system consisting of a powder and a liquid. |
| **Excellent biocompatibility** | confirmed |
| **Adverse Reactions** | According to recent reports, this agent may be associated with adverse reactions including, but not limited to, hypercalcemia, chronic kidney disease, pulmonary embolism, delayed-type hypersensitivity, and even cardiac arrest. |

***Complete set of surgical instruments for vertebroplasty***

| **Device Name** | Spiral Plunger | Puncture Needl |
| --- | --- | --- |
| **Specifications** | 20ml | 2.5×130 |
| **Model** | Model 201 | GC-01 |
| **Registration Certificate Number** | NMPA Authorization No. 201530402 | Shandong MPA Authorization No. 20142140147 |
| **Manufacturer** | Shandong Guanlong Medical Products Co., Ltd. | Shandong Guanlong Medical Products Co., Ltd. |
